# The Singapore Liver Cancer Recurrence (SLICER) Score for Relapse Prediction in Patients with Surgically Resected Hepatocellular Carcinoma

**DOI:** 10.1371/journal.pone.0118658

**Published:** 2015-04-01

**Authors:** Soo Fan Ang, Elizabeth Shu-Hui Ng, Huihua Li, Yu-Han Ong, Su Pin Choo, Joanne Ngeow, Han Chong Toh, Kiat Hon Lim, Hao Yun Yap, Chee Kiat Tan, London Lucien Peng Jin Ooi, Alexander Yaw Fui Chung, Pierce Kah Hoe Chow, Kian Fong Foo, Min-Han Tan, Peng Chung Cheow

**Affiliations:** 1 Division of Medical Oncology, National Cancer Centre Singapore, Singapore, Republic of Singapore; 2 Health Services Research, Singapore General Hospital, Singapore, Republic of Singapore; 3 Centre for Quantitative Medicine, Duke-National University of Singapore Graduate Medical School, Singapore, Republic of Singapore; 4 Department of Pathology, Singapore General Hospital, Singapore, Republic of Singapore; 5 Department of General Surgery, Singapore General Hospital, Singapore, Republic of Singapore; 6 Department of Gastroenterology and Hepatology, Singapore General Hospital, Singapore, Republic of Singapore; 7 Department of Hepatobiliary and Transplant Surgery, Singapore General Hospital, Singapore, Republic of Singapore; 8 Division of Surgical Oncology, National Cancer Centre Singapore, Singapore, Republic of Singapore; 9 Office of Clinical Sciences, Duke-National University of Singapore Graduate Medical School, Singapore, Republic of Singapore; Taipei Veterans General Hosptial, TAIWAN

## Abstract

**Background and Aims:**

Surgery is the primary curative option in patients with hepatocellular carcinoma (HCC). Current prognostic models for HCC are developed on datasets of primarily patients with advanced cancer, and may be less relevant to resectable HCC. We developed a postoperative nomogram, the Singapore Liver Cancer Recurrence (SLICER) Score, to predict outcomes of HCC patients who have undergone surgical resection.

**Methods:**

Records for 544 consecutive patients undergoing first-line curative surgery for HCC in one institution from 1992–2007 were reviewed, with 405 local patients selected for analysis. Freedom from relapse (FFR) was the primary outcome measure. An outcome-blinded modeling strategy including clustering, data reduction and transformation was used. We compared the performance of SLICER in estimating FFR with other HCC prognostic models using concordance-indices and likelihood analysis.

**Results:**

A nomogram predicting FFR was developed, incorporating non-neoplastic liver cirrhosis, multifocality, preoperative alpha-fetoprotein level, Child-Pugh score, vascular invasion, tumor size, surgical margin and symptoms at presentation. Our nomogram outperformed other HCC prognostic models in predicting FFR by means of log-likelihood ratio statistics with good calibration demonstrated at 3 and 5 years post-resection and a concordance index of 0.69. Using decision curve analysis, SLICER also demonstrated superior net benefit at higher threshold probabilities.

**Conclusion:**

The SLICER score enables well-calibrated individualized predictions of relapse following curative HCC resection, and may represent a novel tool for biomarker research and individual counseling.

## Introduction

Hepatocellular carcinoma is often associated with a poor prognosis and is responsible for a disproportionately high global burden of morbidity and mortality. Its incidence is increasing in several developed countries, particularly in Asia as a result of a cohort effect related to infection with hepatitis B and C viruses [1]. To date, surgical resection remains the gold standard treatment in patients with adequate residual liver function, and liver transplant offers the best long term outcomes for patients with impaired liver function secondary to liver cirrhosis. Ablative modalities such as radiofrequency ablation or trans-arterial chemo-embolization are frequently employed for palliative treatment or as a bridge to liver transplant. Despite successful surgical resection and the use of antiviral drugs in the setting of hepatitis-induced liver cirrhosis, the risk of relapse is still extremely high with tumor recurrence developing in up to 70% of cases at 5 years [2].

There have been several scoring systems developed for classification and prognostication of HCC, and these include the American Joint Committee on Cancer staging system 7^th^ edition (AJCC7), Okuda score, Barcelona Clinic Liver Cancer (BCLC), Cancer of the Liver Italian Program (CLIP), Chinese University Prognostic Index (CUPI) and Japan Integrated Staging Score (JIS score) [3–9]. These are predominantly derived from patients with metastatic and locally advanced disease, often with impaired liver function, and have not been validated for use in prediction of relapse after surgical resection. These scoring systems only serve to classify patients into various groups with varying outcomes, but do not predict individualized outcomes. One nomogram based on a smaller dataset in the USA has been proposed to predict disease free survival, and another has been proposed to predict pulmonary metastases, but to date both have not been externally validated [10,11].

From a clinical perspective, there is a need for an accurate model for predicting individualized probabilities of HCC recurrence after curative liver resection. This would guide patient counseling and effective scheduling of clinical surveillance, which is important as early detection of recurrence could be amenable to further curative surgical resection. The model would also help in stratifying patients who may benefit from adjuvant treatment, rank potential liver transplant candidates and serve as a basis for patient selection in clinical trials.

In this study, we have constructed a new postoperative nomogram, the Singapore Liver Cancer Recurrence (SLICER) Score, to predict the probability of freedom from recurrence in patients who have undergone curative surgical resection for HCC. We also demonstrate that it performs better than several major HCC staging systems in use today in predicting probability of freedom from recurrence.

## Patients and Methods

### Ethics Statement

Institutional review board approval from the Singapore Health Services was obtained for this study. All patient records and information was anonymized and de-identified prior to analysis.

### Patients

Patients who underwent primary curative resection for HCC were identified through the hospital database and their medical records were reviewed. We limited our dataset to Singaporean patients who underwent surgery between 1992 and 2007, both to reduce sampling and follow-up bias, as well as to allow for a sufficient duration of post-resection follow-up data to be obtained. All patients underwent a chest x-ray, and either a liver computed-tomography (CT) scan or magnetic resonance imaging (MRI) of the liver prior to surgery. Clinical, radiological and pathological data of these patients were extracted for analysis. The pathological specimens and slides were reviewed by a pathologist specialized in hepatobiliary pathology and tumor characteristics, including but not limited to tumor size, encapsulation, presence or absence of cirrhosis in non-cancerous tissues, resection margin, grade and vascular invasion, were reported. CLIP, CUPI, BCLC, Okuda, Child-Pugh scores and AJCC7 were determined from available data.

All patients were followed up post-operatively according to standard department practices at maximum intervals of 6 months. Clinical surveillance consisted of regular clinical evaluations, serum alpha-fetoprotein (AFP) levels and hepatic imaging in the form of ultrasonography, CT scan or MRI as deemed appropriate by the surgeon. All imaging done was reviewed and reported by radiologists specialized in hepatobiliary imaging. A relapse was diagnosed based on new intra-hepatic or extra-hepatic lesions characteristic of HCC seen on imaging.

### Statistical analysis

The clinical endpoint was freedom from recurrence (FFR). This was defined as the time from date of surgery to date of first relapse detected on imaging, or to the last follow up date for censored cases. A Kaplan-Meier survival curve was used to estimate FFR and univariable Cox regression was carried out to evaluate the effects of various clinicopathologic variables on FFR. Clustering of variables was conducted to reduce the number of potential prognostic factors by evaluating the similarity of these factors [12]. Subsequently, reduced model selection was performed using a backward stepdown by applying the Akaike information criterion [13]. Proportional hazards assumptions were verified systematically for all proposed models. The final multivariable Cox regression coefficients were used to construct the SLICER nomogram. Internal validation was performed to evaluate the ability of SLICER nomogram to predict FFR of individual patients with 200 sets of bootstrap samples as it replicated the development and validation cycle 200 times and used a full or nearly full version of the dataset for each cycle [12]. Calibration plots were drawn to explore the performance characteristics of the nomogram at 3 and 5 years post-resection [12].

Likelihood ratio testing of nested models were performed to compare SLICER to the other prognostic models including CLIP, CUPI, BCLC, OKUDA, AJCC7 and Memorial Sloan-Kettering Cancer Centre (MSKCC) nomogram on a pairwise basis [12]. An adequacy index was used to quantify the percentage of the variation explained by a subset of the individual predictors compared with the information contained in the full set of predictors by means of log-likelihood [12]. Harrell’s c-index was calculated to evaluate the concordance between predicted and observed responses of individual subjects separately [12]. Decision curve analyses, plots of net benefit against threshold probability, were carried out to evaluate these predictive models by examining the theoretical relation between the threshold probability of developing an event and the relative value of false-positive and false negative results as described by Vickers et al [14].

All statistical analyses were done using R 3.0.2 (http://www.R-project.org) and STATA 11 (STATA Corporation, College Station, TX USA), and all tests were two-sided with a significance level of 0.05.

## Results

### Patient characteristics

Between 1992 and 2007, 544 consecutive patients who underwent first-line curative hepatic resection for HCC in Singapore General Hospital were recruited. We excluded foreign patients and patients with missing data (n = 139), leaving 405 patients to form the cohort for our analysis. Their baseline demographic and clinical characteristics are shown in Table 1. The median age of the cohort was 64 years (range 30–88 years), of which 81.7% were male. The cohort consisted predominantly of patients of Chinese ethnicity (92.6%). 62.5% of our patients had either a positive hepatitis B surface antigen, chronic hepatitis C infection, or both. Most patients (89.9%) had biochemical results consistent with Child-Pugh class A status (192 without pathologic cirrhosis), early stage disease (60% with AJCC7 stage I). 265 patients (65.4%) were asymptomatic at diagnosis.

**Table 1:** Baseline demographic and clinical characteristics of 405 patients with resected HCC.

Characteristics	Frequency	(%)
Age (Median (range))	64 (30, 88)	
Tumor size (cm) (Median (range))	4.0 (0.4, 20.0)	
Margin (mm) (Median (range))	3 (0, 10)	
Pre surgery AFP (*ng/mL*) (Median (range))	15.3 (0.8, 70700.0)	
Ethnicity		
Chinese	375	92.6
Malay	11	2.7
Indian	9	2.2
Others	10	2.5
Gender		
Male	331	81.7
Female	74	18.3
ECOG at initiation		
0	240	59.3
1	154	38.0
2	10	2.5
3	1	0.3
Viral hepatitis status		
Hepatitis B	225	55.6
Hepatitis C	25	6.2
Hepatitis B and C	3	0.7
Non hepatitis B/C	129	31.9
Child-Pugh Score		
A	364	89.9
B	41	10.1
Number of nodules		
Single	329	81.4
Multiple	75	18.6
CT lesions location		
Left	60	14.9
Right	328	81.6
Both	11	2.7
Caudate	2	0.5
Diffuse	1	0.2
CLIP		
0	46	11.4
1	236	58.3
2	37	9.1
3	32	7.9
4 to 6	7	1.7
Unquantifiable	47	11.6
CUPI		
Low risk (≤ 1)	341	84.2
Intermediate risk (2–7)	17	4.2
High risk (≥ 8)	0	0.0
Unquantifiable	47	11.6
BCLC		
A	211	52.1
B	29	7.2
C	160	39.5
D	4	1.0
Unquantifiable	1	0.2

### Prognosis

The median follow-up was 25.8 months (range 0.03–173.3 months). A total of222 patients relapsed, and there were 171 deaths. The median overall survival of the cohort was 55.9 months and median FFR was 25.2 months.

### Nomogram construction

**Fig 1:**
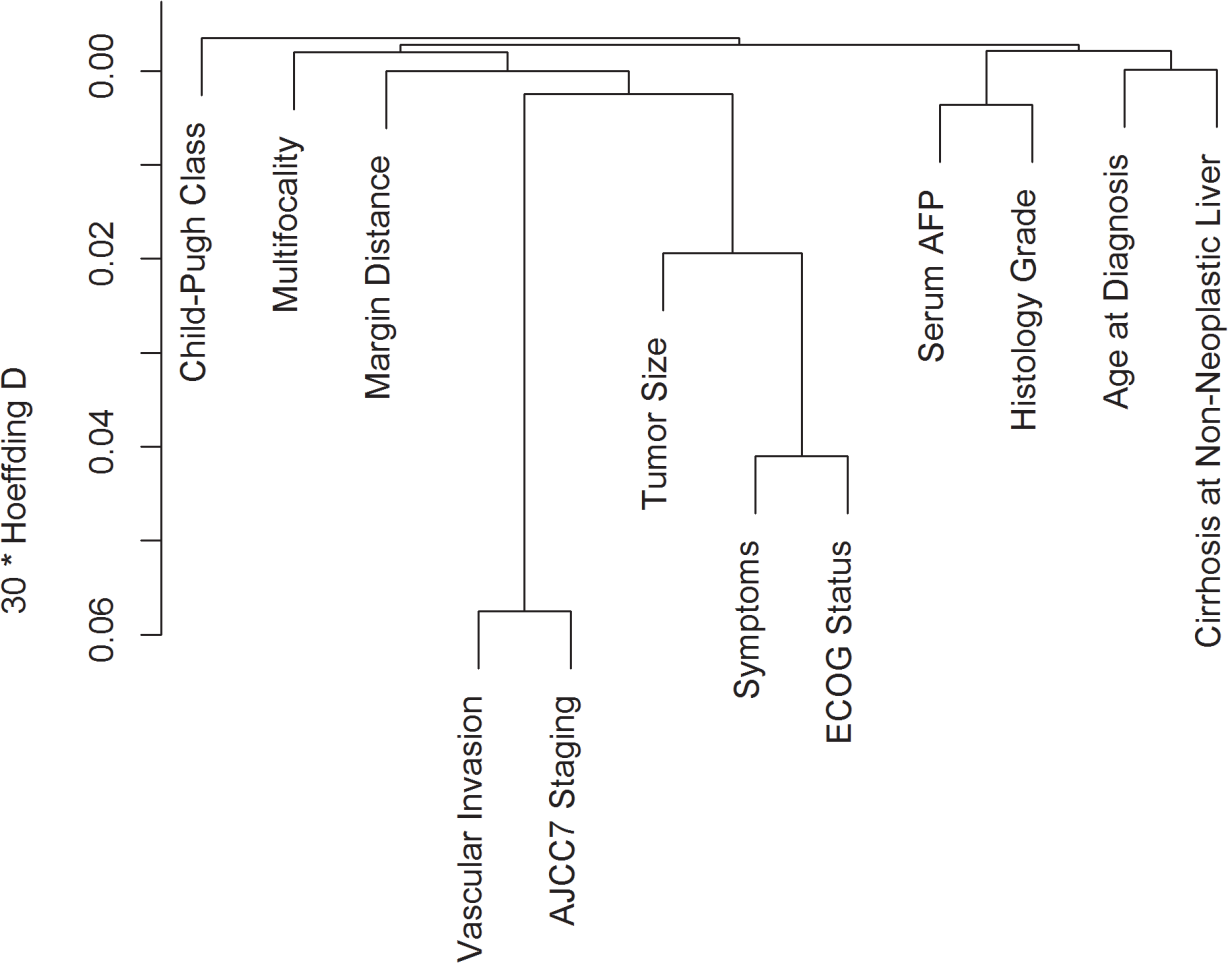
Important clinical variables identified by clustering. These include pre-surgery serum AFP levels, tumor grade, tumor multifocality, tumor margin distance, vascular invasion, AJCC7 Staging, the presence or absence of symptoms at diagnosis, ECOG status, Child-Pugh class status, patient’s age at diagnosis, the presence or absence of cirrhosis in the non-neoplastic liver and tumor size. Hoeffding distance is a ranked based measure of correlation. To illustrate, this figure shows that there is a stronger correlation between vascular invasion and AJCC staging than between serum AFP and tumour grade.

**Fig 2:**
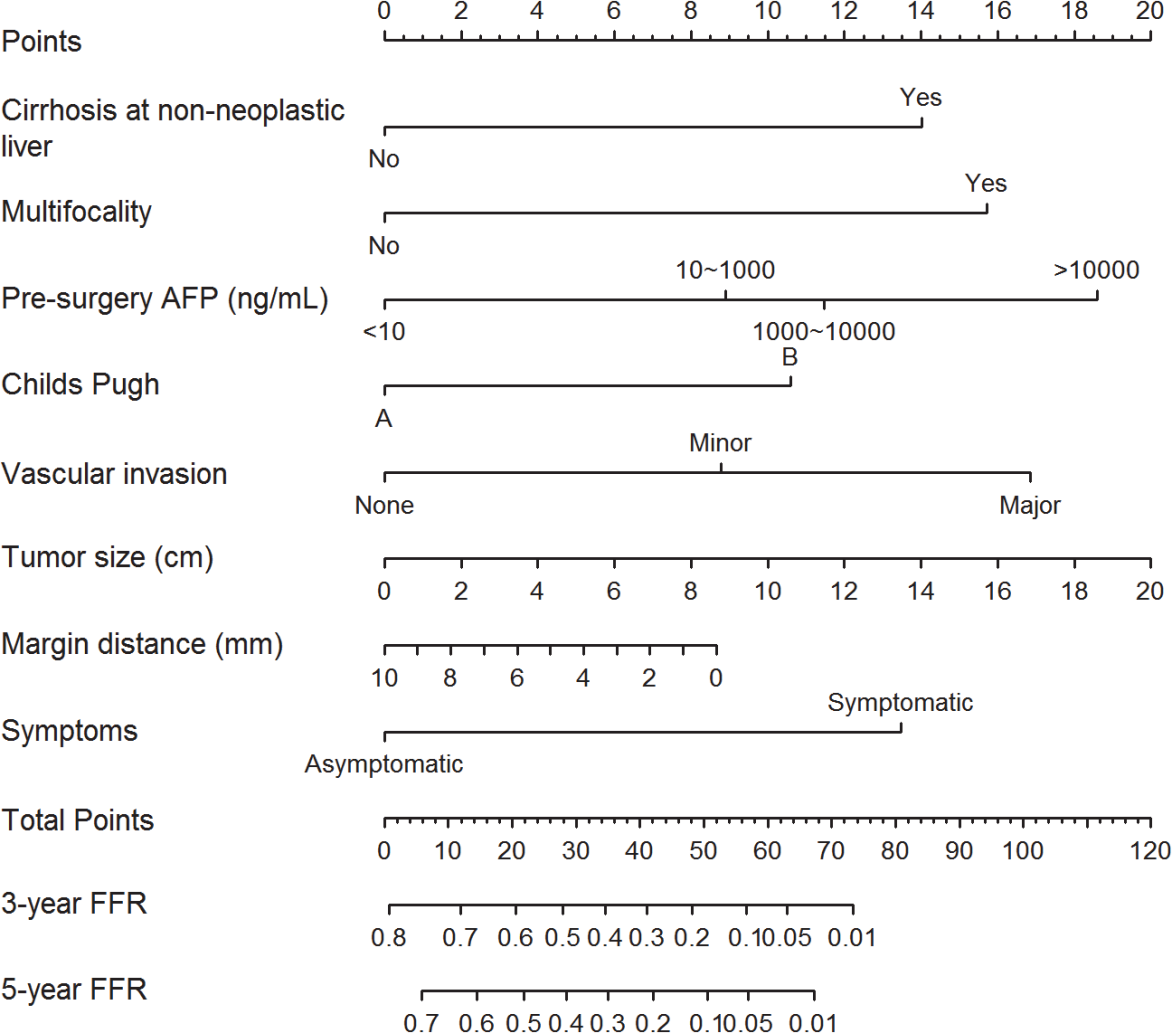
SLICER score nomogram. To use the nomogram, locate the first variable. Draw a line straight upwards to the Points axis to determine the number of points received for the variable. Repeat this process for other six variables and sum up the points achieved for each variable. The sum of these numbers is located on the Total Points axis, and a line is drawn downward to the survival axes to determine the likelihood of 3- or 5-year FFR. For example, a patient who has a 3 cm HCC with multifocality, liver cirrhosis, Child-Pugh A, minor vascular invasion, resection margin 5 mm, pre-surgery AFP 450ng/mL and he was asymptomatic at presentation, total points scored is 48. 3- and 5-years FFR is 16 and 8% respectively.

**Fig 3:**
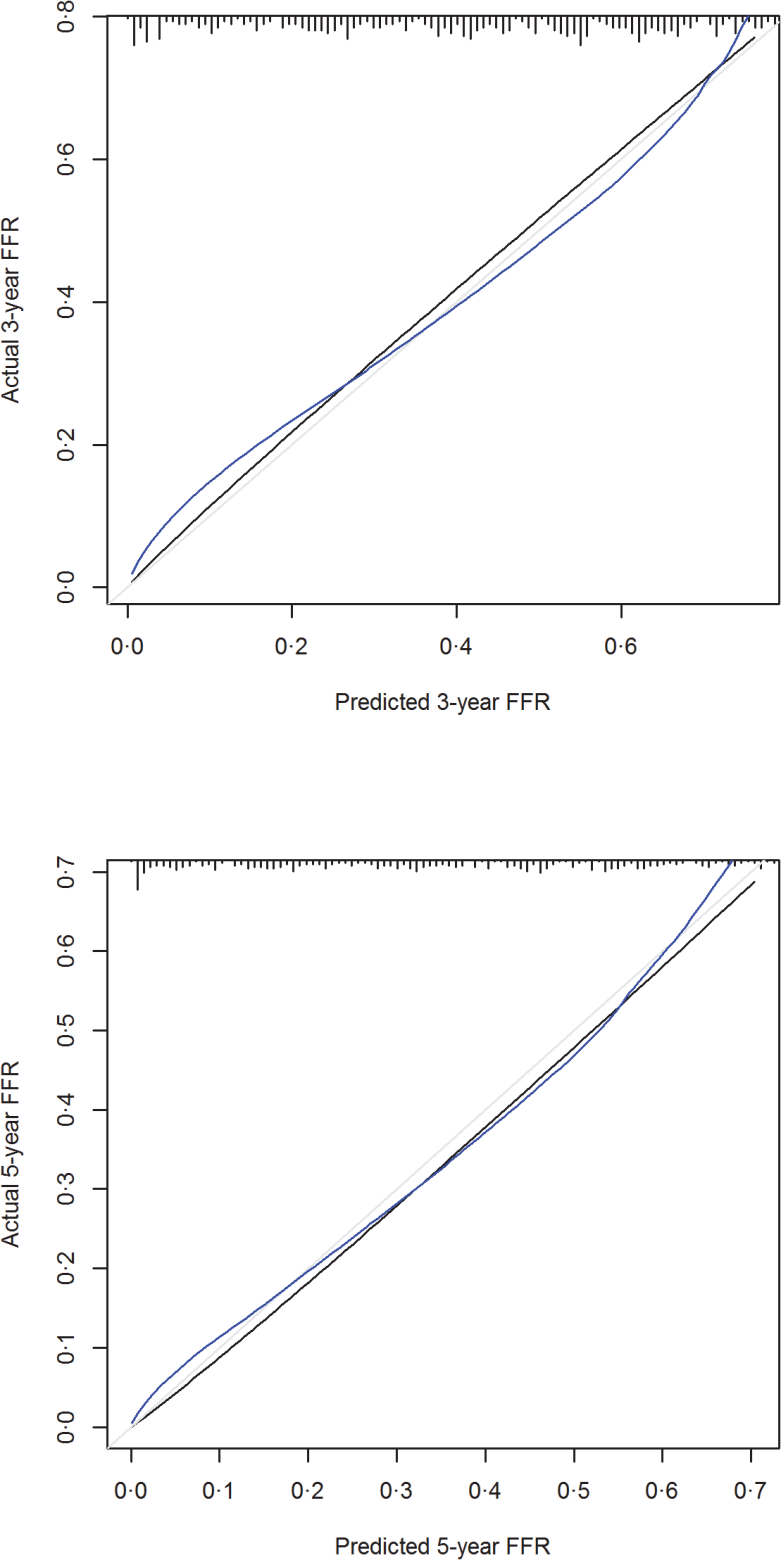
SLICER calibration plots. Plots depict the calibration of SLICER in our dataset in terms of agreement between predicted and observed 3-year and 5-year FFR. Model performance is shown by the plot, relative to the 45° gray line which represents perfect prediction. The black line represents observed outcomes and the blue line represents optimism corrected outcomes generated based on our bootstrap samples.

Univariable analysis showed that Child-Pugh class status, pre-surgery AFP level, non-neoplastic liver cirrhosis, multifocality, vascular invasion, tumor size, margin distance, symptoms, ECOG (Eastern Cooperative Oncology Group) performance status, histological grade and AJCC7 stage significantly affected FFR (Table 2). Among these, ECOG status and AJCC7 stage were excluded from the multivariable analysis as Hoeffding’ D statistics showed that ECOG status and symptoms, vascular invasion and AJCC7 stage were highly correlated (Fig. 1). Finally, 8 of them were found statistically significant in multivariable analysis and selected to construct the nomogram (Table 3, Fig. 2). These include the presence or absence of cirrhosis in the non-neoplastic liver, tumor multifocality, pre-surgery AFP, Child-Pugh class status, vascular invasion, tumor size, margin distance and the presence or absence of symptoms at diagnosis. Bootstrapping was then performed to determine the calibration accuracy for 3-year and 5-year FFR estimates from the final Cox model. Calibration curves were plotted showing that the maximum deviation from ideal were 5.6% and 3.9% respectively (Fig. 3).

**Table 2:** Univariable analysis of variables associated with FFR.

**Variable**	**No. of patients**	**No. of events**	**Median FFR (months)**	**Hazard Ratio (95% CI)**	**P-value**
All	405	222	25.2		
Age	405	222	25.2	1.01 (1.00, 1.02)	0.2393
Cirrhosis at non-neoplastic liver					0.0204
No	192	91	33.7	Reference	
Yes	211	129	21.8	1.37 (1.05, 1.80)	
Satellite nodules or multifocal HCC					<0.0001
No	322	163	30.9	Reference	
Yes	83	59	10.0	2.34 (1.73, 3.17)	
Pre-surgery AFP					0.0001
AFP<10	156	74	33.8	Reference	
10≤AFP<1000	166	97	23.4	1.42 (1.05, 1.92)	
1000≤AFP<10000	31	21	16.9	1.62 (0.99, 2.63)	
≥10000	26	16	7.0	3.28 (1.89, 5.68)	
Child-Pugh Score					0.0004
A	364	194	28.6	Reference	
B	41	28	13.9	2.02 (1.36, 3.02)	
Vascular invasion					<0.0001
None	298	151	33.7	Reference	
Minor	92	60	11.6	2.01 (1.48, 2.72)	
Major	15	11	4.7	3.22 (1.74, 5.96)	
Tumor size	397	217	24.6	1.07 (1.04, 1.10)	<0.0001
Margin distance	396	216	25.2	0.96 (0.93, 1.00)	0.0412
Symptoms					<0.0001
Asymptomatic	265	128	35.2	Reference	
Symptomatic	140	94	13.0	2.14 (1.63, 2.80)	
ECOG					<0.0001
0	240	115	36.4	Reference	
1&2&3	165	107	15.5	1.86 (1.43, 2.43)	
Grade					0.0349
I	50	25	40.0	Reference	
II	170	91	25.0	1.37 (0.87, 2.15)	
III	150	84	23.8	1.60 (1.01, 2.52)	
IV	14	9	10.4	2.87 (1.32, 6.24)	
AJCC7					<0.0001
1	243	112	41.6	Reference	
2	103	63	15.6	1.98 (1.45, 2.70)	
3a	35	29	5.3	4.03 (2.66, 6.10)	
3b	15	11	4.7	3.87 (2.07, 7.20)	
4	9	7	11.6	4.34 (2.01, 9.37)	
Hep B					0.4490
No	171	92	23.8	Reference	
Yes	225	128	25.2	0.90 (0.69, 1.18)	
Hep C					0.0748
No	263	132	28.4	Reference	
Yes	25	16	15.6	1.60 (0.95, 2.69)	

**Table 3:** Multivariable analysis of variables associated with FFR.

**Factor**	**HR (95% CI)**	**p-value**
Cirrhosis at non-neoplastic liver		
No	Reference	
Yes	1.75 (1.27, 2.43)	0.0007
Satellite nodules or multifocal HCC		
No	Reference	
Yes	1.97 (1.44, 2.69)	<0.0001
Pre-surgery AFP		
AFP<10	Reference	
10≤AFP<1000	1.44 (1.06, 1.95)	0.02
1000≤AFP<10000	1.56 (0.98, 2.47)	0.0594
≥10000	1.81 (1.02, 3.22)	0.042
Child-Pugh Score		
A	Reference	
B	1.58 (1.04, 2.39)	0.0324
Vascular invasion		
None	Reference	
Minor	1.47 (1.07, 2.04)	0.0189
Major	1.86 (0.6, 3.62)	0.0662
Tumor size	1.04 (1.00, 1.09)	0.0494
Margin distance	0.96 (0.93, 1.00)	0.0573
Symptoms		
Asymptomatic	Reference	
Symptomatic	1.77 (1.31, 2.38)	0.0002

### Comparison of SLICER to alternative staging models as predictor of FFR

**Fig 4:**
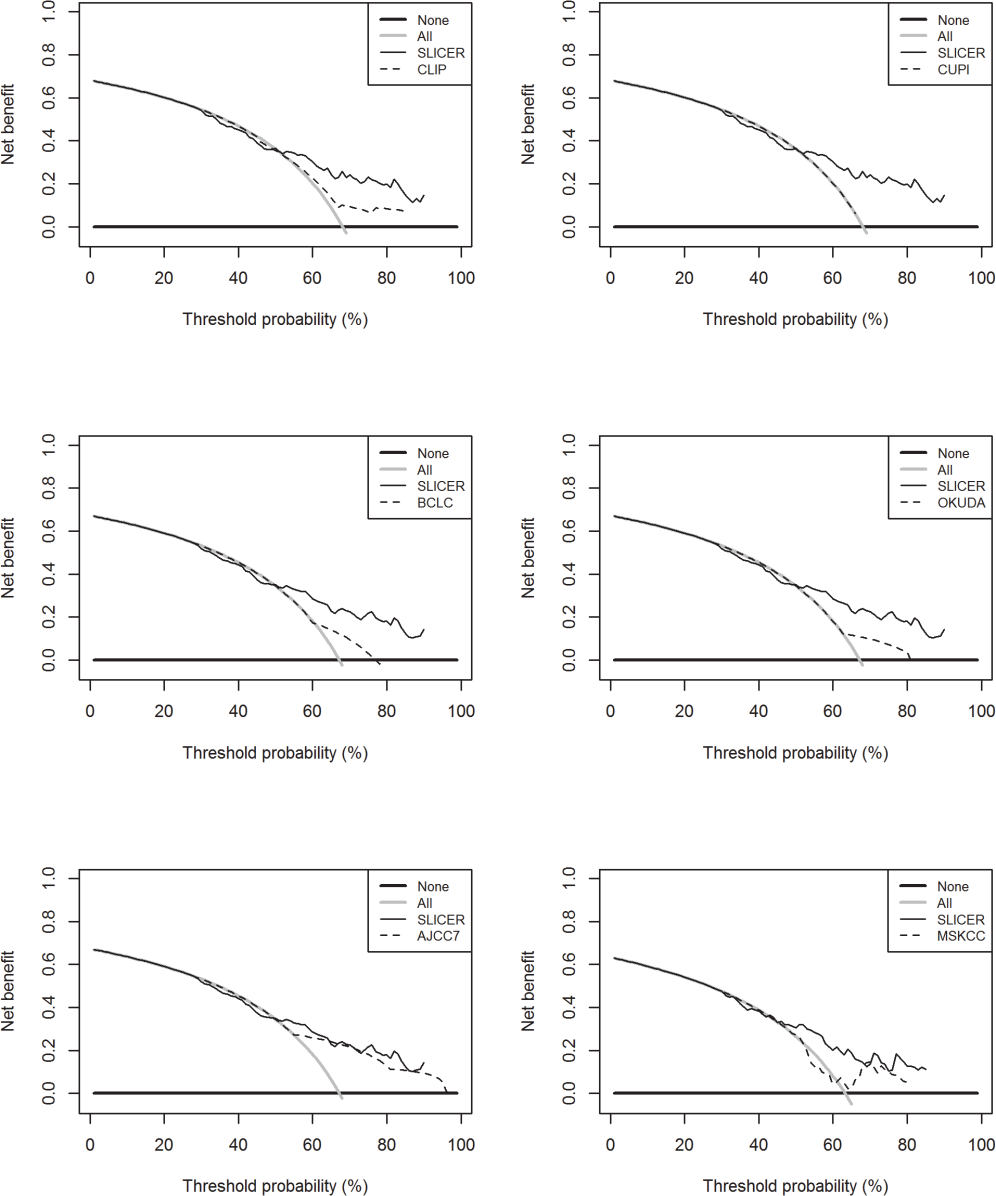
Decision curve analysis. Decision curve analyses depict the clinical net benefit in pairwise comparisons across the different models. SLICER is compared against the various prognostic models in terms of 5-year FFR. Dashed lines indicate the net benefit of SLICER in each of the curves across a range of threshold probabilities. The horizontal solid black line represents the assumptions that no patients will experience the event, and the solid gray line represents the assumption that all patients will relapse. On decision curve analysis, SLICER showed superior net benefit compared to other models across a range of threshold probabilities.

**Fig 5:**
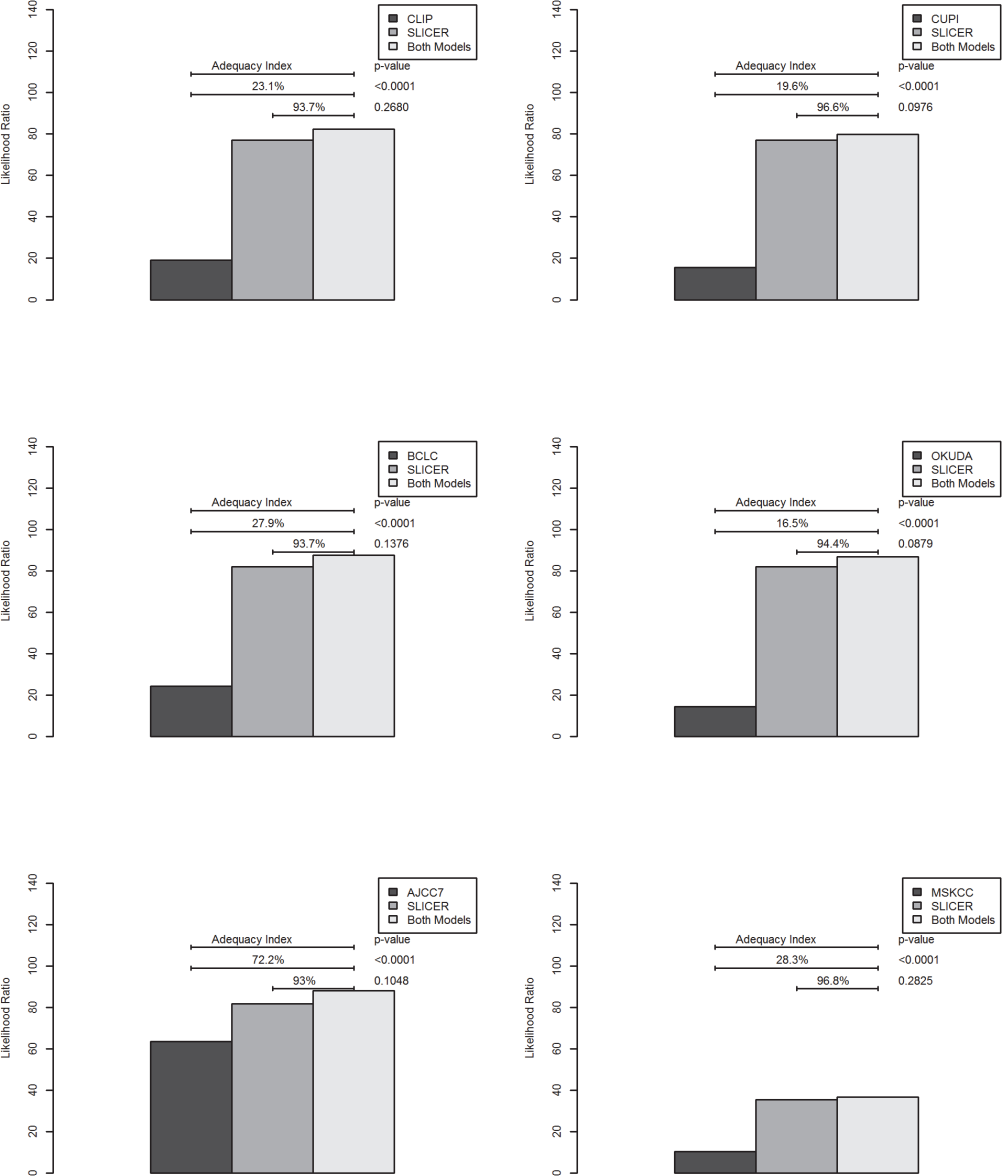
Likelihood analysis. Likelihood analyses compare the SLICER with each of the various models individually, as well as its inclusion into each model, in predicting 5-year FFR. SLICER demonstrated higher adequacy index when compared to each model individually, and its inclusion in each model resulted in highly statistically significant improvements.

The SLICER score demonstrated superior predictive capabilities relative to the other models with a hazard ratio of 1.05 (95%CI 1.04–1.06) and concordance index of 0.69 for prediction of FFR (Table 4). On decision curve analysis [13], compared to other models, our nomogram showed equivalent net benefit between 0–40% threshold probability, but improved performance beyond 40% threshold. This represents superior estimation of decision outcomes at higher threshold probability levels (Fig. 4). The adequacy index of SLICER was also higher when compared to the other models individually. In terms of likelihood analysis, its inclusion in a full model resulted in highly statistically significant improvements when tested with logistic regression analysis against the CLIP (p<0.0001), CUPI (p<0.0001), BCLC (p<0.0001), OKUDA (p<0.0001), AJCC7 (p<0.0001) and MSKCC (p<0.0001) (Fig. 5).

**Table 4:** SLICER, CLIP, CUPI, BCLC, OKUDA, AJCC7 and MSKCC models as predictors of FFR.

Model	Events/ Patients	Hazard Ratio (95% CI)	P-value	5-year FFR	Concordance Index
SLICER	200/366	1.05 (1.04, 1.06)	<0.0001	0.33	0.69
CLIP			<0.0001		0.58
0	16/46	Reference		0.48	
1	133/236	1.84 (1.09, 3.09)		0.34	
2	28/37	3.51 (1.89, 6.50)		0.06	
3	18/32	2.24 (1.14, 4.39)		0.31	
4–6	5/7	6.64 (2.42, 18.23)		NA	
CUPI			<0.0001		0.54
Low Risk (-7–1)	186/341	Reference		0.33	
Intermediate Risk (2–7)	14/17	4.35 (2.50, 7.57)		NA	
High Risk (8–12)	0/0				
BCLC			<0.0001		0.60
A	102/211	Reference		0.39	
B	13/29	1.37 (0.77, 2.45)		0.39	
C	103/160	1.88 (1.43, 2.48)		0.23	
D	3/4	4.31 (1.36, 13.69)		NA	
OKUDA			0.0062		0.54
I	162/307	Reference		0.37	
II	57/94	1.53 (1.13, 2.08)		0.17	
III	3/4	2.76 (0.88, 8.70)		NA	
AJCC7			<0.0001		0.65
1	112/243	Reference		0.34	
2	63/103	1.97 (1.45, 2.70)		0.17	
3	40/50	3.98 (2.76, 5.75)		0.20	
4	7/9	4.34 (2.01, 9.37)			
MSKCC	116/213	1.01 (1.00, 1.01)	0.0003		0.58

## Discussion

HCC is one of the most common malignancies worldwide, especially in Asia in association with a high incidence of Hepatitis B and C infection. Surgical resection offers the best prognosis for long term survival, but little is known about prevention of relapse post-resection and rates of relapse vary over a wide range in published studies. In our centre, we include patients who have an ECOG score of 1, who would otherwise be excluded from surgical resection based on BCLC staging recommendations. This accounts for the high percentage (39.5%) of BCLC stage C patients included in our study.

Several scoring systems for estimation of prognostic outcomes in HCC have been developed to risk stratify patients [15–17], and these systems primarily classify patients in to various risk groups. Nomograms can give each factor a relative weight in prognostication of the outcome, and permit more refined risk estimation for each individual. However, they are challenging to create in a very heterogenous disease such as HCC as many factors need to be considered. In our study, we examined multiple pre-operative and post-operative prognostic factors, and developed a nomogram to quantify expected individualized recurrence outcomes for HCC patients treated by first-line surgical resection. We ensured that these variables are common parameters that are routinely done and easily obtainable for all HCC patients, across all institutions. The SLICER score is meant for use in post-operative patients, and is not meant for pre-operative counseling of the utility of surgery.

To date, one nomogram has been developed for risk estimation of liver cancer recurrence and survival post-hepatectomy at the MSKCC [10]. This nomogram was generated from a small series of 184 patients, and had evident divergence between observed and expected outcomes during calibration, possibly due to the low numbers involved. This nomogram performed less well in our dataset, possibly due to the different patient profile of HCC in the Asian population. In our nomogram, patient age was not a defining variable and the presence of symptoms at diagnosis indicated a higher score. This probably reflects a larger tumor or a later stage tumor at diagnosis, thus translating to an increased risk of relapse.

Risk factors for postoperative recurrence after resection of HCC are related to tumor, host, and surgical factors. Pathologic factors indicative of tumor invasiveness such as vascular invasion, multifocality, large tumor size, and advanced pathological TNM (Tumor, Node, Metastasis) stage are well-established risk factors for recurrence [18]. Microscopic venous invasion as well as macroscopic portal vein involvement are both major risk factors as metastasis via portal venous system is an important mechanism for intrahepatic recurrences [19,20]. The effects of tumor encapsulation and histologic differentiation exert on the risk of recurrence are less conclusive. Active inflammatory activity of the non-neoplastic liver has been associated with a higher proliferative activity and is an independent risk factor for intrahepatic recurrence. Subclinical presentation of HCC is however an independent favorable prognostic factor for disease-free survival [21]. A few studies had identified perioperative blood transfusion as a risk factor for HCC recurrence. Most studies found that the extent of resection and whether anatomical or non-anatomical, had no significant influence on the risk of recurrence. The effect and significance of underlying cirrhosis in the non-neoplastic liver, a wide resection margin to ensure histologic clearance and administration of neo-adjuvant or adjuvant therapy, on the risk of recurrence after resection of HCC, has remained controversial [18]. The results shown in our analysis were consistent with these previous findings.

There has been literature to suggest that early and late recurrence of HCC are thought to be different—early recurrence thought to be a result of metastasis from the same tumor and late recurrence thought to be due to de-novo tumors occurring on a background of field change. The variables we have identified account for both the tumor characteristics (eg. vascular invasion, multifocality, tumor size) and the possible effect of field change (liver cirrhosis, Child-Pugh score), thus accounting for both early and late recurrences of HCC.

More than half our study population (55.6%) are known to be Hepatitis B carriers, reflecting a slightly unique patient profile of HCC patients in Asia. Hepatitis B viral load is known to be an important predictor of HCC recurrence, with most patients being treated with long-term anti-viral therapy as it is thought to reduce HCC recurrence. [22] Unfortunately, in the early part of our study, Hepatitis B viral load was not a routine investigation done for patients and anti-viral therapy was not readily available, thus this variable was not included in our nomogram.

In recent years, molecular research has identified various biomarkers as predictive and prognostic markers for HCC metastatic recurrence and clinical outcomes, including tumor-associated antigens (such as AFP, MAGEs, GPC3, and CK19), molecular factors associated with HCC invasion and metastasis (such as E-cadherin, catenins, ICAM-1, laminin-5), and angiogenesis regulators [23,24]. In particular, elevation of the serum fucosylated fraction of alpha-fetoprotein (AFP-L3) level before treatment is a predictor of HCC recurrence, and sustained elevation of the AFP-L3 level after treatment is an indicator of HCC recurrence [25]. A high pre-operative level of EpCAM-positive circulating tumour cells and some gene expression signatures have also been found to be a predictor for recurrence [26,27]. However the clinical applications and availability of these biomarkers is limited and more extensive studies are needed for validation.

It is useful to note that molecular studies have shown that 60–70% of recurrences are due to true metastasis that results from HCC dissemination before resection [28] and occurs mainly within the first few years after resection [29]. The SLICER score may be predicting a composite of these outcomes. Potential interventions for high-risk patients identified by the nomogram may include systemic and hepatic-artery chemotherapy or chemoembolization; however, these have not been shown to improve overall or disease-free survival after resection of HCC [30]. Early deceased-donor or living-donor liver transplantation is also a consideration. The emergence of targeted therapy such as sorafenib has expanded the scope of therapy in advanced HCC [31,32]. Consequently, there are several ongoing adjuvant clinical trials evaluating agents such as sorafenib and gefitinib for HCC patients after potentially curative treatment [11,33], settings where the SLICER score may be particularly appropriate. While survival remains the main endpoint to measure effectiveness in phase 3 studies, freedom from recurrence is a useful and clinically appropriate endpoint in the adjuvant setting after curative treatment for HCC [34], particularly since repeat resection remains a viable option. A validated nomogram or model therefore is important to assist in patient selection and future clinical trial design and biomarker research.

Our results demonstrate that SLICER outperforms other existing established HCC prognostic models in estimation of FFR, and even more importantly, is well calibrated. Certainly it is true that CLIP, CUPI, BCLC, Okuda scores and AJCC 7^th^ staging systems were developed primarily to predict patient survival rather than relapse. In addition, the data used to develop these models derive mainly from patients with advanced HCC who are not candidates for curative treatment and who generally have poorer liver function. There is thus a clear need for the nomogram we have developed from the largest series to date reported, given the difference in patient profiles between patients with localized and patients with advanced disease. Even more importantly, in addition to discriminatory accuracy, our model was able to provide considerably superior estimation of clinical net benefit at higher threshold probability levels, and comparable estimation of net benefit at lower threshold probability levels, compared to other staging systems. It is important to note that the x-axis of the decision curve analysis diagram is not a direct measurement of model accuracy or performance. It represents a general presentation of estimating decision outcomes (presented as ‘net benefit’) across a range of different risk level thresholds, with two extremes ‘all’ or ‘none’ representing two separate extreme assumptions (‘all’ will relapse and ‘none’ will relapse). It may be seen from the Fig. 4 (top left image) for example that the SLICER and CLIP models showed equivalent net benefit between 0%-40% decision threshold probability, but SLICER showed considerably improved performance over CLIP beyond 40% decision threshold.

Our study indicated that CUPI was least precise for predicting FFR among the 6 staging models tested, with a c-index of only 0.54. CLIP and CUPI staging models have been reported as the most informative regarding survival outcome in advanced HCC [35]. The BCLC classifications stratify HCC into different stages and defines standard of care for each tumor stage. BCLC has been widely used as standard classification for trial design and clinical management of patients with HCC [36]. In our local patient cohort, BCLC does outperform CLIP or CUPI with regard to predicting ability for FFR, but is in turn outperformed by SLICER. Likelihood analysis also showed that inclusion of SLICER in a full model resulted in highly statistically significant improvements when tested with logistic regression analysis against the other models.

Limitations to our study include its retrospective nature, and that it is based on a single-institution experience. As our centre sees approximately 70% to 80% of all surgically resectable patients nation-wide, this may reduce the concern of bias arising from data from a single institution. External validation at other centres will be required. We did not manage to include some other variables known to affect recurrence such as iatrogenic intra-operative tumour rupture [37], or histopathological subtypes of HCC. Fibrolamellar HCC occurs in a distinctly different population of patients than common HCC, and is known to have relatively indolent tumour biology with a better prognosis [38].

Ideally, SLICER should also be paired with a better surveillance model to determine if high-risk patients as identified by SLICER truly benefit from closer surveillance. To date, there are no randomized trials of surveillance strategies in post-operative HCC to determine the benefit of risk-adjusted strategies and SLICER could serve as an appropriate prognostic model for such trials.

To our knowledge, the SLICER score here is developed on the largest reported series of post-surgical patients to date. We have developed a model to predict individualized probabilities of relapse following complete resection of localized hepatocellular carcinoma, in a large, near-national consecutive series of patients, which demonstrates excellent calibration and performance. This provides a foundation for individualized patient counseling and management, biomarker development, and trial design for adjuvant trials in HCC.
