# Correction: The Singapore Liver Cancer Recurrence (SLICER) Score for Relapse Prediction in Patients with Surgically Resected Hepatocellular Carcinoma

**DOI:** 10.1371/journal.pone.0128058

**Published:** 2015-05-13

**Authors:** Soo Fan Ang, Elizabeth Shu-Hui Ng, Huihua Li, Yu-Han Ong, Su Pin Choo, Joanne Ngeow, Han Chong Toh, Kiat Hon Lim, Hao Yun Yap, Chee Kiat Tan, London Lucien Peng Jin Ooi, Peng Chung Cheow, Alexander Yaw Fui Chung, Pierce Kah Hoe Chow, Kian Fong Foo, Min-Han Tan

There is an error in the order of authors. The correct order is: Soo Fan Ang^1^*, Elizabeth Shu-Hui Ng^1^, Huihua Li^2,3^, Yu-Han Ong^1^, Su Pin Choo^1^, Joanne Ngeow^1^, Han Chong Toh^1^, Kiat Hon Lim^4^, Hao Yun Yap^5^, Chee Kiat Tan^6^, London Lucien Peng Jin Ooi^7^, Peng Chung Cheow^7^, Alexander Yaw Fui Chung^7^, Pierce Kah Hoe Chow^8,9^, Kian Fong Foo^1^, Min-Han Tan^1^*

**1** Division of Medical Oncology, National Cancer Centre Singapore, Singapore, Republic of Singapore, **2** Health Services Research, Singapore General Hospital, Singapore, Republic of Singapore, **3** Centre for Quantitative Medicine, Duke-National University of Singapore Graduate Medical School, Singapore, Republic of Singapore, **4** Department of Pathology, Singapore General Hospital, Singapore, Republic of Singapore, **5** Department of General Surgery, Singapore General Hospital, Singapore, Republic of Singapore, **6** Department of Gastroenterology and Hepatology, Singapore General Hospital, Singapore, Republic of Singapore, **7** Department of Hepatobiliary and Transplant Surgery, Singapore General Hospital, Singapore, Republic of Singapore, **8** Division of Surgical Oncology, National Cancer Centre Singapore, Singapore, Republic of Singapore, **9** Office of Clinical Sciences, Duke-National University of Singapore Graduate Medical School, Singapore, Republic of Singapore.
